# Primary Remitting Seronegative Symmetrical Synovitis With Pitting Oedema: A Case Report

**DOI:** 10.7759/cureus.77570

**Published:** 2025-01-17

**Authors:** Inês Pintor, Martina Arandjelovic, João Oliveira, Leonor Naia, Rosa Jorge

**Affiliations:** 1 Internal Medicine, Hospital Infante D. Pedro, Aveiro, PRT; 2 Infectious Disease, Hospital Infante D. Pedro, Aveiro, PRT

**Keywords:** arthritis, oedema, rs3pe, seronegative, synovitis

## Abstract

Remitting seronegative symmetrical synovitis with pitting oedema (RS3PE) syndrome is a rare disease that typically presents in older men and is characterised by bilateral and symmetrical peripheral arthritis, pitting oedema in the dorsal hands and feet, and seronegativity for rheumatoid factor. A notable feature of this syndrome is its excellent response to systemic corticosteroid therapy.

This article presents a case of a 75-year-old White male patient recently diagnosed with bilateral carpal tunnel syndrome. He reported complaints of distal and symmetrical inflammatory polyarthralgia for three months, morning stiffness lasting 10 minutes, bilateral hand muscle weakness, and bilateral oedema in the hands and feet. Upon examination, the patient exhibited pain during the palpation of the bilateral metacarpophalangeal, proximal interphalangeal, and tibiotarsal joints, all of which also exhibited signs of inflammation; similarly, flexor tenosynovitis was identified in both hands. Laboratory tests revealed anaemia, hypoalbuminaemia, and elevated acute phase reactants, with negative autoimmune screening and rheumatoid factor. X-rays showed osteoarthritis with decreased periarticular bone density and no evidence of erosions. The patient underwent active screening for neoplasia, which was not evident.

A diagnosis of RS3PE syndrome was established and the patient initiated systemic corticosteroids, gradually tapered over a year, with a favourable response. However, due to symptom recurrence, corticosteroid therapy was reintroduced and continued for an additional two years, resulting in complete and sustained remission.

With this case report, the authors emphasize a rare clinical entity that often mimics other rheumatological disorders, which must be ruled out during the diagnostic process. A high degree of clinical suspicion is essential for diagnosis, after which active screening for neoplasia is required, since RS3PE frequently occurs as a paraneoplastic syndrome.

## Introduction

Remitting seronegative symmetrical synovitis with pitting oedema (RS3PE) syndrome is a rare condition, typically occurring in older men. It is characterised by the acute onset of bilateral and symmetrical peripheral arthritis, pitting oedema in the dorsal hands and feet, and seronegativity for rheumatoid factor [1-4]. The aetiology remains unclear and it may present as a primary condition or in association with viral infections, medications or vaccines, or even have a paraneoplastic origin [4-7].

This case report discusses a 75-year-old male patient diagnosed with primary RS3PE syndrome who achieved symptom remission after initiating corticosteroid therapy but required a longer tapering period.

## Case presentation

This article presents the case of a 75-year-old White male patient evaluated in an outpatient Internal Medicine appointment for complaints of sudden onset of distal and symmetrical inflammatory polyarthralgia, with a three-month progression. Concomitantly, the patient reported morning stiffness lasting 10 minutes, an inability to perform a pincer grasp in both hands, bilateral pitting oedema in the hands and feet, as well as paraesthesia in the upper limbs, in the median nerve distribution. There was no improvement after a week of etoricoxib 90 mg, taken once daily.

The patient’s medical history included essential hypertension, dyslipidaemia, benign prostatic hyperplasia, and a recent diagnosis of bilateral carpal tunnel syndrome. Regular medication comprised tamsulosin+dutasteride 0.4+0.5 mg once daily, atorvastatin 20 mg once daily, and imidapril 20 mg once daily.

During the appointment, the patient reported no additional complaints, including shoulder and pelvic girdle muscle weakness, weight loss, anorexia, fever, nocturnal hyperhidrosis, gastrointestinal or genitourinary symptoms, cough, dyspnoea, or Raynaud’s phenomenon. There was no recent introduction of new medications or vaccinations at the onset of symptoms, and there was no history of recent viral infection.

Upon physical examination, notable findings included apyrexia, mucocutaneous pallor, bilateral pitting oedema in the dorsal hands and feet, severe tenderness on palpation of the bilateral metacarpophalangeal, proximal interphalangeal, and tibiotarsal joints, all of which also exhibited signs of inflammation; similarly, flexor tenosynovitis was identified in both hands. No other abnormalities were observed, specifically no joint deformities or nodules or cutaneous changes. Laboratory results revealed normocytic and normochromic anaemia (haemoglobin 10.7 g/dL; reference range 13-18 g/dL), an elevated erythrocyte sedimentation rate (ESR) (71 mm; reference range <15 mm), a raised C-reactive protein (CRP) (16.73 mg/dL; reference range <0.5 mg/dL), hypoalbuminaemia (3.05 g/dL; reference range 3.4-4.8 g/dL), hyperferritinaemia (613 ng/mL; reference range 22-322 ng/mL), and increased alpha-1 and alpha-2 globulins on serum protein electrophoresis.

The remaining laboratory investigations were unremarkable, including hepatic enzyme levels, renal and thyroid function, and procalcitonin. Testing for autoimmune diseases was negative (rheumatoid factor; anti-citrullinated peptide antibodies; antinuclear antibodies; anti-deoxyribonucleic acid (anti-DNA), anti-Sjögren’s syndrome-related antigen A (anti-SSA), anti-Sjögren’s syndrome-related antigen B (anti-SSB), anti-histone, anti-Smith antigen (anti-SM), and anti-ribonucleoprotein (anti-RNP) antibodies). Immunoglobulin levels were normal. Screening for syphilis was negative, as were serological tests for human immunodeficiency virus (HIV), hepatitis B, and hepatitis C. The interferon-gamma release assay (IGRA) and blood cultures were also negative.

**Figure 1:**
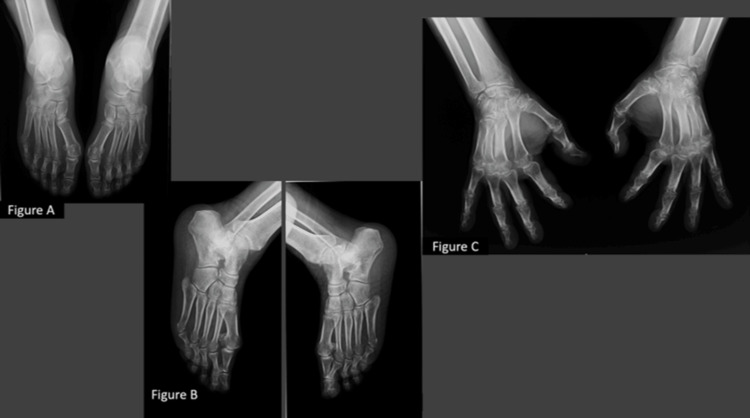
X-rays of the hands and feet X-rays of the hands and feet show reduced periarticular bone density. Figure C also demonstrates soft tissue oedema.

Regarding imaging, X-rays of the hands and feet demonstrated reduced periarticular bone density, but no deformities or erosions (Figure 1).

RS3PE syndrome was assumed and the patient was started on prednisolone 20 mg once daily, resulting in rapid clinical improvement, including resolution of the oedema and joint pain. Laboratory markers also improved, with haemoglobin increasing to 12.4 g/dL, ESR reducing to 4 mm, CRP to 0.25 mg/dL, and ferritin to 315 ng/mL.

Further investigations included thoraco-abdomino-pelvic and cranioencephalic computed tomography (CT) scans, which did not show any features of neoplasia. Upper gastrointestinal endoscopy and colonoscopy revealed evidence of diffuse chronic gastritis, mild intestinal metaplasia, and an ulcerated antral polyp (12 mm) which was excised and sent for histopathological examination, confirming it as a hyperplastic polyp.

Prednisolone was gradually tapered over a year, with a favourable response and the patient remained asymptomatic during this period. Approximately three months after discontinuation, the patient experienced a recurrence of symptoms, including inflammatory arthralgia and bilateral oedema in the feet. Prednisolone 20 mg was reintroduced which resolved the symptoms and an even slower steroid-tapering regimen occurred over two years.

Currently, the patient maintains follow-up appointments, remaining asymptomatic, without any evidence of neoplasia and there has been no need for corticosteroid reintroduction in the last four years. A recent screening showed no evidence of cancer.

## Discussion

RS3PE syndrome is a rare rheumatological disorder, first described in 1985 by McCarty et al. as seronegative rheumatoid arthritis of the elderly, characterised by a benign course and oedema in the dorsum of the hands and feet [1]. It predominantly occurs in elderly patients and is more common in men [1,3], consistent with the clinic case presented here.

Its aetiology remains uncertain, although there have been reports of associations with viral infections, medications, vaccines, and neoplasia, often manifesting as a paraneoplastic syndrome, particularly in the haematological, digestive tract, gynaecological, and urological cancers [3-7]. Genetic predisposition has also been described, particularly in individuals who are HLA-B7 [8] and HLA-A2 positive [9]. In this case report, the patient showed no evidence of viral infection and had no history of recent introduction of new medications or vaccinations. At the time of diagnosis and over the subsequent four years of follow-up, there was no evidence of neoplasia, which led to the conclusion of primary RS3PE syndrome.

Clinical manifestations have a sudden onset, typically within 24 to 48 hours, and include symmetrical distal polyarthritis accompanied by pain and functional limitation, primarily affecting the metacarpophalangeal, proximal interphalangeal and carpal joints, and less commonly the shoulders, knees, ankles and feet. Other features include extensor and flexor tenosynovitis of the hand, and pitting oedema in the dorsum of the hands and feet due to venous obstruction, increased capillary permeability, lymphatic obstruction, and distal synovitis [1,10-12].

Some patients may present with bilateral carpal tunnel syndrome, associated with oedema and carpal region tenosynovitis. There have also been reports of hepatic and cutaneous involvement [12].

Our patient exhibited the main clinical features of this syndrome, although without arthritis of the shoulders or knees, which is less frequently reported. He also presented bilateral carpal tunnel syndrome.

Laboratory findings typically show elevated acute-phase markers; hypoalbuminaemia and anaemia are also common. In some cases, there may be a rise in transaminases. Leucocytosis is uncommon. Rheumatoid factor is usually negative. HLA-B7 is positive in approximately half of the cases, and antinuclear antibodies may be positive at a low titre [3,12,13]. This patient presented with elevated inflammatory markers, hypoalbuminaemia, and anaemia, with negative rheumatoid factor and antinuclear antibodies.

In 1994, Olivo et al. proposed diagnostic criteria, which include over 65 years of age, negative rheumatoid factor, symmetrical polysynovitis (affecting the wrists, metacarpophalangeal, and proximal interphalangeal joints), pitting oedema, morning stiffness, rapid response to corticosteroid therapy, and exclusion of other diseases [14].

Regarding differential diagnosis, it is crucial to exclude at least three clinical entities in older adults: (1) Rheumatoid arthritis: more common in women between the ages of 30 and 70, characterised by symmetrical and distal polyarthritis, without bilateral oedema, with positive rheumatoid factor in 80% of cases, typical radiographic findings, and has a favourable response to corticosteroids. (2) Polymyalgia rheumatic: more frequent in women between the ages of 70 and 80, involving the pelvic and shoulder girdles, with bilateral oedema in up to 12% of cases, and highly responsive to corticosteroids. (3) Late-onset spondyloarthritis: more prevalent in men in their 60s, involving the knees, hips, and ankles and has poor to no response to corticosteroid therapy [12,15].

RS3PE syndrome generally follows a short and benign course and is highly responsive to low-dose corticosteroid therapy. Prednisolone, at a dose of 15 to 20 mg daily, is the most commonly used treatment. The majority of patients report improvement within 24 to 72 hours after starting treatment. Once clinical presentation and laboratory results have improved, a gradual corticosteroid taper is initiated and treatment duration typically ranges from 6 to 18 months [12]. The reported recurrence rate is 9%, and only a few isolated cases of corticosteroid resistance have been described [12].

Our patient demonstrated an excellent initial response to treatment, with tapering over a year. However, following discontinuation, symptoms recurred, necessitating a new course of corticosteroid therapy and an even slower taper over two years.

## Conclusions

The case presented here is the characteristics of RS3PE syndrome, fulfilling the diagnostic criteria. This is a rare condition, with few reported cases, and often poses a diagnostic challenge due to clinical overlap with other autoimmune rheumatological disorders. It typically has a benign course and is highly responsive to corticosteroid therapy, with no lasting sequelae. Despite this, most RS3PE cases occur in the setting of a paraneoplastic syndrome, meaning that neoplasia must be ruled out at the time of diagnosis.

This case report aims to raise awareness of this rare, and often underdiagnosed, clinical entity and its broad differential diagnoses. One of the major obstacles to diagnosis is maintaining high clinical awareness, since once RS3PE is suspected, establishing the diagnosis is not particularly challenging. Another difficulty lies in ruling out more severe conditions, such as neoplasia, which may not be present initially but can emerge later. This highlights the importance of long-term surveillance.
